# Changes in the pharmacokinetic of sildenafil citrate in rats with Streptozotocin-induced diabetic nephropathy

**DOI:** 10.1186/2251-6581-13-8

**Published:** 2014-01-07

**Authors:** Alok S Tripathi, Papiya M Mazumder, Anil V Chandewar

**Affiliations:** 1 Department of pharmacology, P. Wadhwani college of Pharmacy, Yavatmal, Maharashtra 445001, India; 2 Department of Pharmaceutical sciences, Birla Institute of technology, Mesra, Ranchi, Jharkhand 835215, India; 3 Deparment of Pharmaceutical chemistry, P. Wadhwani college of Pharmacy, Yavatmal, Maharashtra 445001, India

**Keywords:** Diabetic nephropathy, Streptozotocin, Sildenafil citrate

## Abstract

**Aim:**

The present investigates deals with the change in the pharmacokinetic of Sildenafil citrate (SIL) in disease condition like diabetic nephropathy (DN).

**Method:**

Diabetes was induced in rats by administering Streptozotocin i.e. STZ (60 mg/kg, IP) saline solution. Assessment of diabetes was done by GOD-POD method and conformation of DN was done by assessing the level of Creatinine, Blood Urea Nitrogen (BUN) and Albuminurea. After the conformation of DN single dose of drug SIL (2.5 mg/kg, *p.o.*) were given orally and Pharmacokinetic Parameters like [AUC o-t (ug.h/ml), AUC 0-∞, C_max_, T_max_, Kel, Clast] were estimated in the plasma by the help of HPLC-UV.

**Result:**

There was significant increase (p < 0.01) in the Pharmacokinetic parameters of SIL in DN rat (AUC_0-t_, AUC_0-∞_, C_max_, T_max_ and T_1/2_) compare to normal control rat and significant increase Kel in the DN rat compare to control rat.

**Conclusion:**

The study concluded that there was significant (p < 0.01) increase in the bioavailability of SIL in DN.

## Introduction

Diabetes mellitus is a complex endocrine metabolic disorder. Globally, as of 2010, an estimated 285 million people had diabetes, with type 2 making up about 90% of the cases [1]. Its incidence is increasing rapidly, and by 2030, this number is estimated to almost double [2]. India has more diabetics than any other country in the world, according to the International Diabetes Foundation [3]. Clinically, the main consequence of hyperglycemia is secondary impairments in various tissues and organs. Some complications such as cardio vascular disease, kidney disease, retinopathy, and nervous system diseases result in substantial morbidity and mortality [4]. What is especially note worthy that the pharmacokinetic parameters of many drugs are altered in diabetes [5-8]. The possible reasons for these pharmacokinetics changes are diverse and complex, including gastrointestinal lesions which cause changes in drug absorption [9]; changes in transporters responsible for uptake, efflux and elimination [10]; changes in liver drug enzymes which alters metabolic rate [11]; nephropathy which leads to changes of drug transport, metabolism and elimination [12]. However, the pharmacokinetic parameters of drugs are usually determined from healthy subjects. In clinical studies it has been reported that drug accumulated in some diabetic patients [13], and diabetes may influence pharmacodynamics and increase the adverse effect of hypoglycemic agents. Therefore, the study of drug pharmacokinetics in the diabetic state is important and beneficial for clinical development.

Diabetic nephropathy (DN) is a progressive, irreversible disease characterized by increasing blood pressure, microalbuminuria, proteinuria, and a continuous decline in glomerular filtration rate [14]. There was number of drugs used in the management of DN. Sildenafil citrate (SIL), 1-[4-ethoxy-3-(6, 7-dihydro-1-methyl-7-oxo-3-propyl-1*H*- pyrazolo [4, 3-*d*] pyrimidin-5-yl) phenylsulfonyl] - 4 methylpiperazine, primarily indicated in the treatment of erectile dysfunction [15]. It acts by inhibiting cGMP-specific phosphodiesterase type 5, an enzyme that promotes degradation of cGMP, which regulates blood flow in the penis. Literature suggest phosphodiesterase inhibitors type 5 (Sildenafil citrate) having role in the management Diabetic Nephropathy So it is worthy to check the change in the pharmacokinetics of SIL in Diabetic complication like DN [16].

## Material and methods

### Animal

Healthy Male Sprague–Dawley rats (200–300 gm) were used for the pharmacological screening. The animals were housed in polypropylene cages with wire mesh top and husk bedding and maintained under standard environmental conditions (25 ± 20C, relative humidity 60 ± 5%, light- dark cycle of 12 hours each) and fed with standard pellet diet (Trimurti feeds, Nagpur) and water ad libitum, were used for the entire animal study. The rats were housed and treated according to the rules and regulations of CPCSEA and IAEC. The protocols for all the animal studies were approved by the Institutional Animal Ethical Committee (IAEC). CPCSEA registration no.- (650/02/C/CPCSEA/08).

### Instrumentation

A double beam UV-Visible spectrophotometer, model UV-2401 PC (Japan) with 10 mm matched quartz cell was used.

The HPLC instrument consisted of Thermo separation product quaternary gradient equipped with pump spectra system P-4000 having inline membrane degasser. Detector was a UV visible detector belonging to spectra system UV 1000. Rheodyne 9725 injector with 20 μl loop. All the data was processed using Data Ace software. Separation was achieved using a Prontosil C18 stationary phase (150 7× 4.6 mm i.d. 5 μm particle size) and The analytical column was protected by a Phenomenex C18 guard column (4 mm × 2*.*0 mm, i.d.).

### Materials and reagents

Sildenafil citrate was donated by Ajanta Pharmaceuticals pvt. ltd. All the reagent and chemical used were of AR analytical & HPLC grade. Methanol (Spectrochem) and water (Lobachem) used were of HPLC grade.

### Induction of diabetes

Diabetic rats were induced with an ip injection of 60 mg/kg of STZ (dissolved in pH 4.5 citrate buffer immediately before injection), while controlled normal rats (Control group, *n* = 6) received 2.5 mL/kg of citrate buffer. Induction of the diabetic state was confirmed by measuring the blood glucose level at the 72 h after the injection of STZ. Rats with more than 200 mg/dl Blood glucose level was conformation of diabetes. Blood glucose level and biomarkers of nephropathy were checked after 21 day for the conformation of Nephropathy. After the conformation of Diabetic Nephropathy single dose of drugs were given orally to the following groups and the blood glucose level were checked. Group I: Control group + SIL (2.5 mg/kg), Group II: Negative Control (STZ) + SIL (2.5 mg/kg).

### Pharmacokinetic study

Single dose of SIL citrate was given orally to different groups of rat for Pharmacokinetic studies. The rats were fasted overnight with free access to water before administration of drugs. After a single oral administration of SIL (2.5 mg/kg, p.o.), 0.5 ml of blood samples were collected from retro orbital plexus sinus at 0.5, 1, 2, 4, 6, 12 and 24 h time-points. Plasma was separated by centrifugation and stored at -20°C until analysis. Aliquots of 0.1 ml serum samples were processed on the developed HPLC Method which was developed in our lab [17].

The pharmacokinetic parameters were calculated with a Non-Compartmental model using Kinetica TM Soft-ware (version 4.4.1 Thermo Electron Corporation, U.S.A). Each value is expressed as Mean ± SD.

### Statistical analysis

Data were analyzed using Graph Pad Prism version 5.0 for Windows (Graph Pad Software, San Diego, CA, USA). Data were statistically analyzed using Student *t* test.

## Result

Table 1 shows that there was significant increase (p < 0.01) in the blood glucose level (289.66 ± 3.40), BUN (20.39 ± 1.52) and creatinine (1.255 ± 0.07) level in blood in STZ treated rat as compare to Control group. Whereas STZ treated group shows significant increase (p < 0.01) Albumin urea (266.7 ± 0.20) level in the urine compare to control group. These results confirm the nephropathy induction.

**Table 1:** Effect of STZ on blood glucose level, Serum Creatinine, BUN and Albuminurea level in rats

**Sr. no.**	**Groups**	**Blood glucose level (mg/dl)**	**Serum creatinine (mg/dl)**	**BUN level (mg/dl)**	**Albuminurea (mg/day)**
1	Control	108.00 ± 2.26	0.750 ± 0.07	10.89 ± 2.64	97.75 ± 0.25
2	Negative control (STZ)	289.66 ± 3.40^@^	1.255 ± 0.07^@^	20.39 ± 1.52^@^	266.7 ± 0.20^@^

Data are the mean ± SEM for 6 rats,

^@^p < 0.01 compare to normal Control.

**Figure 1:**
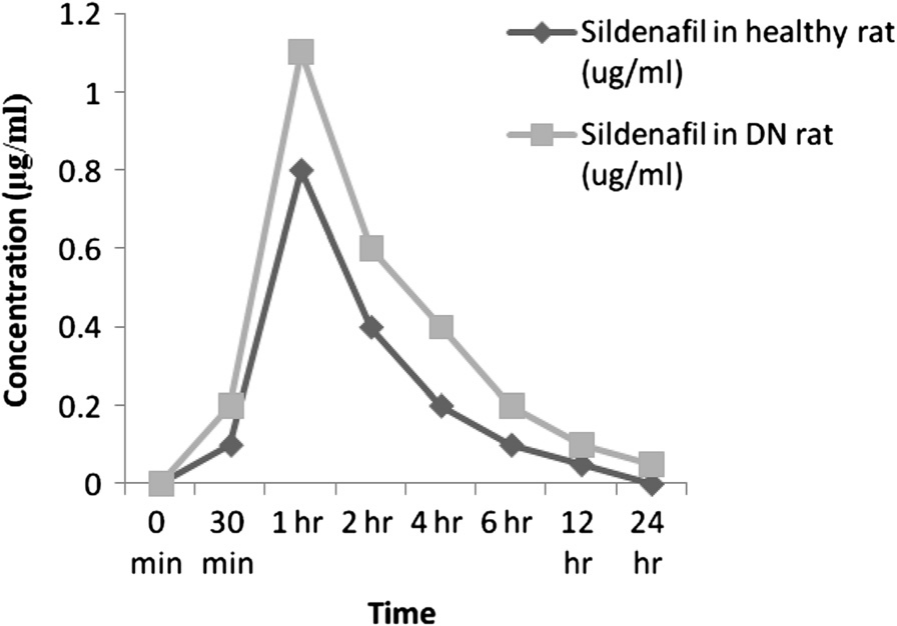
Mean serum concentration-time profile of SIL after oral administration of 2.5 mg/kg of SIL in rats.

Table 2 shows that the effect of Diabetic Nephropathy on pharmacokinetic of SIL. The Pharmacokinetic parameters like AUC_0-t_, AUC_0-∞_, C_max_, T_max_, Kel and T_1/2_ were assessed. There were significant increase (p < 0.01) in the Pharmacokinetic parameters of SIL in DN rat (AUC_0-t_, AUC_0-∞_, C_max_, T_max_ and T_1/2_) compare to normal control rat and significant increase Kel in the DN rat compare to control rat. Figure 1 shows the serum concentration-time profile of SIL after oral administration of 2.5 mg/kg of SIL in rats.

**Table 2:** Pharmacokinetic variables of SIL after oral administration at the dose of 2.5 mg/kg to control rats and diabetic rat

**Sr. no.**	**Group**	**AUCo-t (ug.h/ml)**	**AUC 0-∞ (ug.h/ml)**	**C max (ug)**	**Tmax (hr)**	**Kel**	**T**_**1/2**_
1	Control + SIL (2.5 mg/kg)	2.19 ± 0.052	0.5 ± 0.02	0.8 ± 0.05	1 hr	0.10 ± 0.01	6.93
2	STZ + SIL (2.5 mg/kg)	3.72 ± 0.098*	1.12 ± 0.09*	1.1 ± 0.1*	1 hr^ns^	0.089 ± 0.002*	8.6*

Data are the mean ± SEM for 6 rats,

^*^p < 0.01 compare to normal Control.

## Discussion

In the present study type 1 diabetes was induced by the STZ in experimental rats [18] and the AUC and C max of SIL were compared with control rat. Diabetic Nephropathy was marked by increase in the Serum creatinine, Blood urea nitrogen in blood and albumin urea in urine [19]. In the present study diabetic nephropathy was conformed as there were significant increases in these values.

Sildenafil citrate is widely used as selective inhibitors of cyclic guanosine monophosphate (cGMP)-specific phosphodiesterase type 5 (PDE5) inhibitors in the treatment of erectile dysfunction (ED) [20,21]. They can also be efficient as therapy for a range of cardiovascular diseases, such as pulmonary arterial hypertension (PAH) [22-24]. The major route of elimination of sildenafil is hepatic metabolism, with renal excretion of unchanged drug [25].

Diabetic patients have higher level of circulating glucose in the blood, leading to non-enzymatic glycation of several proteins including albumin. Glycated albumin exhibits atherogenic effects in various cells [26]. Non-enzymatic glycation of albumin produces conformational changes in the structure of albumin (affinity of the phenytoin binding site on albumin based on a modification of the lysine group) [27], which can increase the free fraction of acidic drugs in patients with type 1 and 2 diabetes (for more detail, see Table two) [28-35]. Worner et al. [28] reported a 50% decrease in binding of dansylsarcosine to albumin in diabetic patients, whereas the concentration of circulating albumin was the same in diabetic patients [36,37]. Glycation of blood and plasma proteins leads to reduction in protein binding capacity [38-40]. A linear relationship has been reported between the degree of albumin glycation and the unbound fraction of drug in the serum of diabetic patients. Thus, for highly albumin bound acidic compounds the reduction in the plasma serum protein binding capacity has been shown in diabetic patients [41].

In DN and Control rat single dose of SIL was given orally, and different pharmacokinetic parameters were assessed. As DN leads to decrease in the GFR [42] and protein content in the blood as there is microalbuminurea in DN. This may increase the bioavailability of SIL in DN rat. There was (Table 2) increase in the pharmacokinetic parameters like AUC0_-t_, AUC_0-∞_, C_max_, T_max_, Kel and T_1/2_ in DN rat compare to normal control rat.

## Conclusion

Increase in the pharmacokinetic parameters of SIL confirms its increased bioavailability in DN rats.

## Competing interests

The authors declare that they have no competing interests.

## Authors’ contributions

Dr. PMM: Performed the analysis of data, drafting and editing, Dr. AVC: Supervision, proof read and edited the manuscript. AST: principle investigator, wrote the proposal, conducted the study and did the initial drafting. All authors read and approved the final manuscript.
